# A study on the Neotropical Anthaxiini (Coleoptera, Buprestidae, Buprestinae)

**DOI:** 10.3897/zookeys.304.5313

**Published:** 2013-05-22

**Authors:** Svatopluk Bílý

**Affiliations:** 1 Czech University of Life Sciences, Faculty of Forestry and Wood Sciences, Department of Forest Protection and Entomology, Kamýcká 1176, Praha 6 – Suchdol, CZ-165 21, Czech Republic

**Keywords:** Taxonomy, Coleoptera, Buprestidae, Buprestinae, Anthaxiini, Anthaxiina, new genera, new subgenera, new species, new combinations, lectotype designation, key, Neotropical region

## Abstract

Revision of the Neotropical genera of the subtribe Anthaxiina Gory & Laporte, 1839 (Coleoptera, Buprestidae, Buprestinae, Anthaxiini). Five new genera are described: *Anthaxita* **gen. n.**, *Charlesina* **gen. n.**, *Cobosina* **gen. n.**, *Marikia* **gen. n.** and *Sanchezia* **gen. n.** Genus *Agrilaxia* Kerremans, 1903 is divided into two subgenera: *Agrilaxia* and *Costiptera* **subgen. n.** and the genus *Bilyaxia* Hołyński, 1989 is divided into three subgenera: *Bilyaxia*, *Paraguayetta* **subgen. n.** and *Tomasia* **subgen. n.** One new species is described: *Anthaxita peruviana* **sp. n.**, and two informal species-groups are suggested within *Agrilaxia* (*Costiptera* **subgen. n.**): *Agrilaxia (Costiptera) modesta* (Kerremans, 1897) species-group and *Agrilaxia (Costiptera) occidentalis* (Kerremans, 1900) species-group. Lectotype is designated for *Agrilaxia mrazi* Obenberger, 1932. A key of all genera/subgenera is provided and all treated taxa are illustrated.

## Introduction

During a recent study on the Neotropical genus *Agrilaxia* Kerremans, 1903 (Bílý & Brûlé, in press) I have found some taxonomic problems concerning also the related Neotropical genera of the tribe Anthaxiini Gory & Laporte, 1839. Some time ago I suggested a new concept of this group (Bílý 2004) which was also followed by Bellamy (2008). After separating off several genera to the subtribe Curidina Hołyński, 1989 (Bílý 2004), the subtribe Anthaxiina Gory & Laporte, 1839 contained only 10 genera: *Afragrilaxia* Bílý & Bellamy, 1999, *Agrilaxia* Kerremans, 1903, *Anthaxia* Eschscholtz, 1829, *Bilyaxia* Hołyński, 1989, *Brachanthaxia* Théry, 1930, *Brachelytrium* Obenberger, 1923, *Brasilaxia* Théry, 1935, *Chalcogenia* Saunders, 1871, *Paracuris* Obenberger, 1923 and *Tetragonoschema* Thomson, 1857. After upgrading the subgenus *Bilyaxia* to genus level (Bílý 2004) and after the definition of the genera *Ctenoderus* Germain, 1856, *Cylindrophora* Solier, 1849 and *Romanophora* Bílý, 2004 and the transfer of these genera to the subtribe Curidina (Bílý 2004), some species of *Anthaxia* subgen. *Cylindrophora* sensu Cobos, 1956 and 1975 were automatically and formally transferred to the genus *Bilyaxia*. The final, 11th genus was added to Anthaxiini after the revision of *Tetragonoschema* by the upgrading of the subgenus *Anilaroides* Théry, 1934 to genus level (Bílý, 2012). The situation is clarified in the present paper.

As mentioned above, only 11 genera remain in the subtribe Anthaxiina, 6 of them belonging to the Neotropical fauna: *Agrilaxia*, *Anilaroides*, *Bilyaxia*, *Brasilaxia*, *Paracuris* and *Tetragonoschema*. The genera *Brasilaxia* and *Paracuris* are monotypic taxa without the taxonomic problems, the genus *Agrilaxia* was revised by Cobos (1972) and the genera *Anilaroides* and *Tetragonoschema* by Bílý (2012). Since all species of *Anthaxia* subgen. *Cylindrophora* sensu Cobos, 1956, 1972 and 1975 (except for those which were transferred to the different genera of Curidina) were formally attributed to *Bilyaxia* (Bílý, 2004), the genus became rather heterogenous containing the following species: *Bilyaxia auronotata* (Bílý, 1978), *Bilyaxia bruchiana* (Obenberger, 1928), *Bilyaxia bucki* (Cobos, 1956), *Bilyaxia cinctipennis* (Kerremans, 1913), *Bilyaxia concinna* (Mannerheim, 1837), *Bilyaxia cordillerae* (Obenbeger, 1928), *Bilyaxia cupriceps* (Fairmaire & Germain, 1858), *Bilyaxia descarpentriesi* (Cobos, 1956), *Bilyaxia emmanueli* (Cobos, 1972), *Bilyaxia macullicollis* (Kerremans, 1887), *Bilyaxia mariae* (Cobos, 1956), *Bilyaxia mrazi* (Obenberger, 1932), *Bilyaxia obscurata* (Reed, 1873), *Bilyaxia rubricollis* (Moore, 1981) and *Bilyaxia willineri* (Cobos, 1972). The enigmatic species, *Anthaxia paulseni* Fairmaire & Germain, 1860 most probably does not belong to Anthaxiini; the type is probably lost and according to the very brief description, this species looks like some species of Acmaeoderini Kerremans, 1893 (Cobos, 1956). Last but not least, also the large genus *Agrilaxia* had to be split to two subgenera and some species were transferred to *Bilyaxia*.

## Material and methods

The morphological terms specific for the Neotropical Anthaxiini follow Cobos (1972) and Bílý and Brûlé (in press).

A Canon D-550 digital camera with the Canon MP-E65 mm f/2.8 1-5× macro lens was used to capture the colour images.

Data from locality labels are cited „verbatim“ with my comments in [square brackets], individual labels are indicated by a double slash („//“); p= printed, h= handwritten.

**ISNB** Institut Royal des Sciences Naturelles, Brussels, Belgium

**MFCB** Museum Frey, Basel, Switzerland

**MNCN** Museo Nacional de Ciencias Naturales, Madrid, Spain

**NMPC** National Museum, Prague, Czech Republic

**USNM** United States National Museum of Natural History, Smithsonian Institution, Washington D.C., U.S.A.

**ZIN** Zoological Institute, Russian Academy of Sciences, St. Petersburg, Russia

## Results

The Neotropical Anthaxiini differ by some characters from the Old World representatives of the tribe and some of them are, without any doubt, of Gondwanian origin (see also Cobos, 1958). Some morphological features are characteristic only of the Neotropical species, e.g. the “agriloid” pronotal carina and subhumeral, elytral carina (Fig. 23 in Bílý & Brûlé, in press), simply ocellate pronotal sculpture, usually asetose body (with some exceptions in *Agrilaxia*, *Tetragonoschema* and in *Sanchezia* gen. n.), and usually simply spindle-shaped male genitalia without the lateral serrations of the median lobe (except for some species of *Tetragonoschema* with spines on parameres). Other typical characters of the Neotropical Anthaxiini are the prolonged scutellum which is usually distinctly longer than wide (Figs 31, 32), unarmed male metatibiae, brush-like row of dense bristles on inner margin of protibiae (Fig. 33) and the form of prosternum in some *Agrilaxia* and *Bilyaxia* (Fig. 34); the specific morphological characters of the genus *Agrilaxia* are discussed in Cobos (1972) and Bílý & Brûlé (in press).

### Key separating the genera of the Neotropical Anthaxiina

**Table:** 

1	Body flat, Lycid-like; antennae robust, antennomeres 3–10 triangular to trapezoidal, pedicel triangular; frons and ventral surface with rather long, white pubescence; elytral epipleura wide, enlarged posteriorly, reaching elytral apex; elytra flat, without transverse, basal depression; prescutellar pit missing; lateroposterior pronotal depressions very wide, deep, reaching anterior fourth of pronotum; humeral swellings small, not projecting beyond outline of elytra; larger species (5.7–7.0 mm); Fig. 5; Argentina, Brasil	*Sanchezia* gen. n.
–	Body moderately or strongly convex, *Anthaxia* or *Agrilus*-like (e.g. Figs 19–25) or flat, wide, with shortened, uneven elytra (e.g. Figs 26–28); antennae not conspicuously enlarged, antennomeres 4–10 triangular to trapezoidal, pedicel suboval or subcylindrical; lateroposterior pronotal depressions missing or well defined, shallow or deep but never reaching anterior pronotal fourth; basal, transverse, elytral depression well defined; humeral swellings well defined, usually projecting beyond outline of elytra	2
2	Body moderately or strongly convex, (e.g. Figs 19–25); entire body asetose (except for a few species of *Agrilaxia* and *Tetragonoschema* with ventral pubescence); elytral epipleura narrower, often nearly missing, never wide or enlarged posteriorly; prescutellar pit well defined, sometimes deeply punctiform; basal, transverse elytral depression complete, reaching scutellum; elytra glabrous or with more or less defined longitudinal costae	3
–	Body flattened or weakly convex, elytra usually short, uneven, with wide, flat depressions, 1.1–2.3 times as long as wide (Figs 26–28); at least frons and ventral surface with white pubescence (often also pronotum and elytra with short pubescence); apex of each elytron widely, regularly rounded; elytral epipleura wide, subparallel, reaching elytral suture; anal ventrite situated vertically or nearly vertically; entire anal tergite (pygidium) clearly visible from above; sculpture of basal portion of elytra the same as that on pronotum	13
3	Body prolonged, *Agrilus*-like (Figs 19–25); pronotum more than 1.4 times as wide as long; elytra usually more than 2.5 times as long as wide (except for *Agrilaxia* subgenus *Costiptera* subgen. n. – see below); posterior margin of pronotum (usually hidden under base of elytra) straight, only exceptionally serrate; lateroposterior pronotal depressions usually well defined; prescutellar pit and basal, pronotal tubercles on both sides of pit mostly well defined; anal ventrite and tergite sometimes sharply serrate or with needle-like spines	12
–	Body not prolonged, more or less *Anthaxia*-like (Figs 10–18); pronotum 1.5–2.0 times as wide as long; elytra less than 2.5 times as long as wide; anal tergite usually simply rounded without conspicuous serrations; lateroposterior pronotal depressions missing or weakly defined; prescutellar pit and basal, pronotal tubercles usually missing or only weakly defined	4
4	Body wedge shaped; frons deeply, widely, longitudinally grooved; pronotum regularly narrowing anteriorly, without lateroposterior depressions and “agriloid” carina; pronotal sculpture consisting of very dense, small, deep, sometimes nearly punctiform, polygonal cells; elytra with 3 complete longitudinal costae and well defined, strongly elevated subhumeral carina, reaching elytral apex; 6.2–7.0 mm; Fig. 16; Brasil	*Brasilaxia* Théry, 1935
–	Body not wedge shaped, more or less *Anthaxia*-like; frons convex, flat or moderately depressed; pronotal margins subparallel or rounded, maximum pronotal width at anterior third or at midlength; lateroposterior pronotal depressions and “agriloid” carina well defined or missing; pronotal sculpture consisting of fine polygonal cells (usually with lustrous or shagreened bottom) or very fine, transverse rugae; elytra without longitudinal costae, rarely with 2–3 weakly defined, longitudinal elevations; subhumeral carina weakly defined, sometimes almost missing	5
5	Body unicoloured, bronze; pronotum more or less convex, lateroposterior depressions and “agriloid” carina missing or almost indistinct; pronotal sculpture very fine, consisting of subtle polygonal cells with microsculptured bottom or by almost indistinct transverse rugae or transversely widened, poorly visible cells; suture between abdominal ventrites 1–2 very weakly defined or invisible; elytra covering entire abdomen	6
–	Body multicoloured, rarely entire body golden green;pronotum with black, medial, usually wide stripe reaching both anterior and posterior margins or with two longitudinal stripes; lateroposterior, pronotal depressions wide, “agriloid” carina well defined; pronotal sculpture consisting of well defined polygonal cells; suture between abdominal ventrites 1–2 well defined; elytra somewhat shortened, anal tergite (pygidium) visible from above or elytra conspicuously shortened, posterior 3 tergites not covered by elytra	9
6	Frons strongly convex; clypeus very short, transverse, with straight anterior margin; pronotum convex, “agriloid” carina and lateroposterior pronotal depressions very weakly defined; pronotal sculpture consisting of well defined polygonal cells with shagreened bottom; elytral apices widely, regularly rounded; elytral epipleura very narrow but reaching nearly apex of elytra; subhumeral carina almost indistinct, elytral sculpture relatively rough consisting of simple punctures and fine rugae; 4.2–4.4 mm; Fig. 3; Argentina, Brasil	*Cobosina* gen. n.
–	Frons flat or widely grooved; clypeus trapezoidal, slightly emarginate anteriorly; pronotum weakly convex or flattened with wide, shallow lateroposterior depressions or depressions missing; pronotal sculpture poorly visible, consisting of weakly defined polygonal cells or pronotum without clearly defined sculpture; elytral apices narrowly rounded, sometimes almost caudiform; elytral epipleura narrow, reduced, reaching at most posterior third of elytral margins; subhumeral carina usually well defined; elytral sculpture very fine	7
7	Frons with wide, medial groove; pronotum flat with wide, shallow lateroposterior depressions, without distinct sculpture, matt; elytra 2.3 times as long as wide; subhumeral carina well defined, long, reaching apical portion of elytra; elytral sculpture consisting of fine microsculpture and very small, lustrous grains; 6.2 mm; Fig. 2; Brasil	*Charlesina* gen. n.
–	Frons flat or very slightly convex; pronotum moderately convex, without lateroposterior depressions; pronotal sculpture consisting of very fine, weakly defined, polygonal cells; elytra 1.8–2.0 times as long as wide; elytral sculpture consisting of fine punctures and transverse rugae	8
8	Posterior pronotal angles obtuse, lateral carina reaching only posterior third of lateral margin; prosternal process subparallel; posterior margin of pronotum (covered by elytral base) serrate; terminal antennomere slightly longer than wide; body matt, ventral surface with rather long, sparse, white pubescence; 5.4 mm; Fig. 1; Peru	*Anthaxita* gen. n.
–	Posterior pronotal angles rectangular, lateral carina reaching pronotal midlength; prosternal process slightly widened behind procoxae; posterior margin of pronotum (covered by elytral base) straight; terminal antennomere 2.5 times longer than wide; body lustrous, ventral surface asetose; 4.0–5.5 mm; Fig. 4; Ecuador	*Marikia* gen. n.
9	Elytra shortened, last 3 tergites clearly visible from above (but usually covered by hind wings); pronotum 1.5 times as wide as long, with very rough, ocellate sculpture; elytral sculpture consisting of rather large, sparse grains, each elytron with 2–3 more or less defined, longitudinal costae; both anal ventrite and tergite with roughly serrate posterior margins; subhumeral carina reduced, short, weakly defined; 8.0–9.0 mm; Figs 17, 18; Argentina, Bolivia	*Paracuris* Obenberger, 1923
–	Elytra slightly shortened, only anal tergite (pygidium) visible from above; pronotum 1.6–2.3 times as wide as long with fine, polygonal cells (usually rougher along lateral margin); elytra without longitudinal costae or exceptionally with 2–3 fine, obsolete costae; elytral sculpture consisting of fine punctures and short transverse rugae or tiny, lustrous grains on microsculptured backround; only anal ventrite with serrate (sometimes very finely) posterior margin; subhumeral carina well defined, long	10
10	Body wide, flat or moderately convex; frons flat or slightly depressed; pronotum 2.0–2.3 times as wide as long; lateroposterior depressions wide, shallow or completely missing; pronotum sometimes with weak, medial, longitudinal groove; elytra 1.8–2.0 times as long as wide, without any traces of longitudinal costae	11
–	Body prolonged, subcylindrical; frons convex; pronotum 1.6–1.7 times as wide as long; lateroposterior depressions wide, rather deep; elytra 2.4 times as long as wide with subhumeral carina nearly reaching elytral apex; elytra smooth or with more or less defined, longitudinal costae; 3.2–5.0 mm; Figs 13–15; Argentina, Uruguay	*Bilyaxia* (*Tomasia* subgen. n.)
11	Frons flat, very slightly convex or finely grooved; pronotal sculpture consisting of well defined, polygonal cells; lateroposterior depressions shallow, wide, more or less fusing to each other or nearly indistinct; subhumeral carina well defined but not sharp; prescutellar pit missing; prosternum flat of slightly convex; antennomeres 5–10 as wide as long; smaller species (3.7–6.5 mm); Figs 10, 11; Chile	*Bilyaxia* (subgen. *Bilyaxia*)
–	Frons rather deeply grooved; pronotum densely shagreened with rather indistinct, poorly defined, polygonal sculpture; lateroposterior depressions missing; subhumeral carina long, elevated, reaching elytral apex; prescutellar pit deep; antennomeres 5–10 distinctly wider than long; prosternum transversely grooved just behind anterior margin which is elevated, forming transverse lamina perpendicular to prosternal plate (like in Fig. 34e); larger species (7.5 mm); Fig. 12; Paraguay	*Bilyaxia* (*Paraguayetta* subgen. n.)
12	Body strongly prolonged, *Agrilus*-like, elytra more than 2.5 times as long as wide; subhumeral carina shorter, reaching elytral midlength, sometimes very short, exceptionally missing; elytra smooth or with more or less defined, flat, longitudinal costae; prescutellar pit and basal, pronotal tubercles on both sides of pit usually well defined; ”agriloid” carina exceptionally missing; anal ventrite (sometimes also tergite) finely to strongly serrate; uni- to multicoloured species; 3.6–10.0 mm; Fig. 19–25; from U.S.A. to Patagonia	*Agrilaxia* (subgen. *Agrilaxia*)
–	Body strongly wedge shaped, not very elongate; elytra less than 2.5 times as long as wide; subhumeral carina strongly defined, reaching elytral apex; elytra with 2–3 well defined, longitudinal costae; prescutellar pit shallow, basal pronotal tubercles on both sides of pit weak to missing; “agriloid” carina very well defined, reaching anterior third of pronotum; anal ventrite finely serrate, anal tergite usually without lateral serrations; entirely green-bronze or blue-bronze species; 5.0–7.2 mm; Fig. 7–9; Argentina, Brasil	*Agrilaxia* (*Costiptera* subgen. n.)
13	Bodyelongate, more *Anthaxia*-like; elytra 1.9–2.3 times as long as wide, covering entire abdomen including pygidium, regularly, weakly convex or slightly depressed behind scutellum; anal ventrite more or less horizontal; elytral epipleura narrower, not reaching elytral suture; elytra with conspicuous mirror-effect along posterior half of suture (as in *Anilara* Saunders, 1868); Fig. 26; Brasil	*Anilaroides* Théry, 1934
–	Body conspicuously shortened, sometimes nearly as wide as long (Figs 27–28); elytra 1.1–1.4 times as long as wide, usually strongly uneven with several wide, deep depressions; entire pygidium visible from above, anal ventrite nearly vertical; elytral epipleura wide, reaching or nearly reaching elytral suture; elytra without distinct mirror-effect	14
14	Body black, bronze or multicoloured, shortened (sometimes nearly as wide as long) species; elytra usually very short, flat, conspicuously uneven with one common, medial depression and with deep lateral and preapical depressions; frons usually deeply impressed with projecting prominences above antennal insertions, rarely frons convex; pronotum less convex, transverse, usually with well defined lateroposterior depressions; antennae and tarsi black; Fig. 27; from Mexico to Argentina	*Tetragonoschema* subgen. *Tetragonoschema* Thomson, 1857
–	Body dark bronze, subcylindrical; elytra completely or partly red-bronze; elytra convex, without lateral and preapical depressions only with flat, medial, triangular depression at anterior elytral third; frons convex with weak postclypeal depression; pronotum regularly, rather strongly convex; antennae and tarsi reddish-brown; Fig. 28 southern Argentina	*Tetragonoschema* subgen. *Patagoschema* Bílý, 2012

**Figures 1–6.**
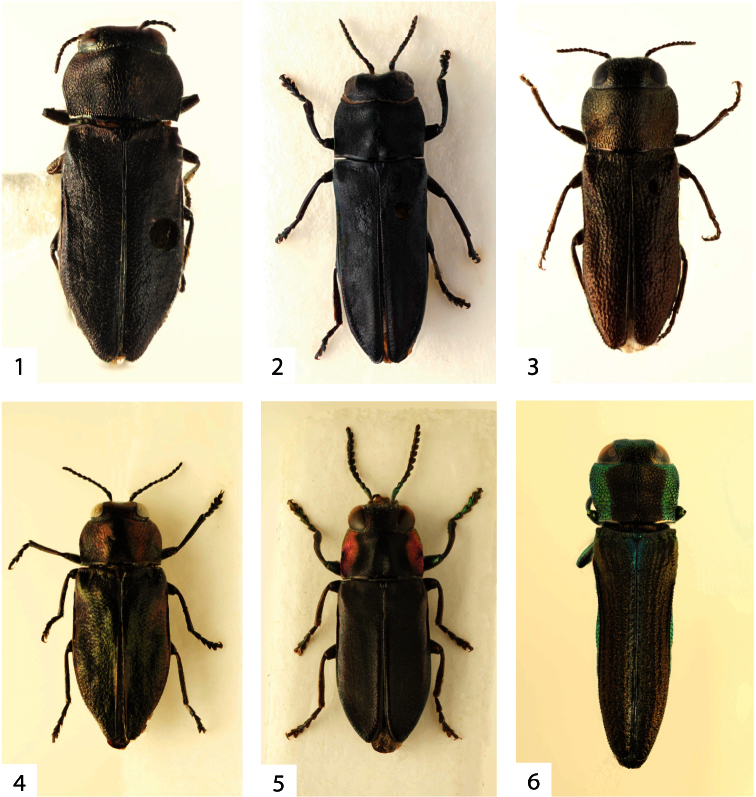
**1** *Anthaxita peruviana*, sp. n., female holotype, 5.4 mm **2** *Charlesina mrazi mrazi* (Obenberger, 1932), male lectotype, 6.2 mm **3** *Cobosina willineri* (Cobos, 1972), male, 4.4 mm (Brasil, Catamarca) **4** *Marikia descarpentriesi* (Cobos, 1956), male, 4.9 mm (Ecuador, Pichincha) **5** *Sanchezia bucki* (Cobos, 1956), 7.0 mm (male holotype of *Cylindrophora kafkai* Bílý, 1996) **6** *Agrilaxia (Agrilaxia) flavimana* (Gory, 1841), male, 3.8 mm (USA, Arizona).

**Figures 7–12.**
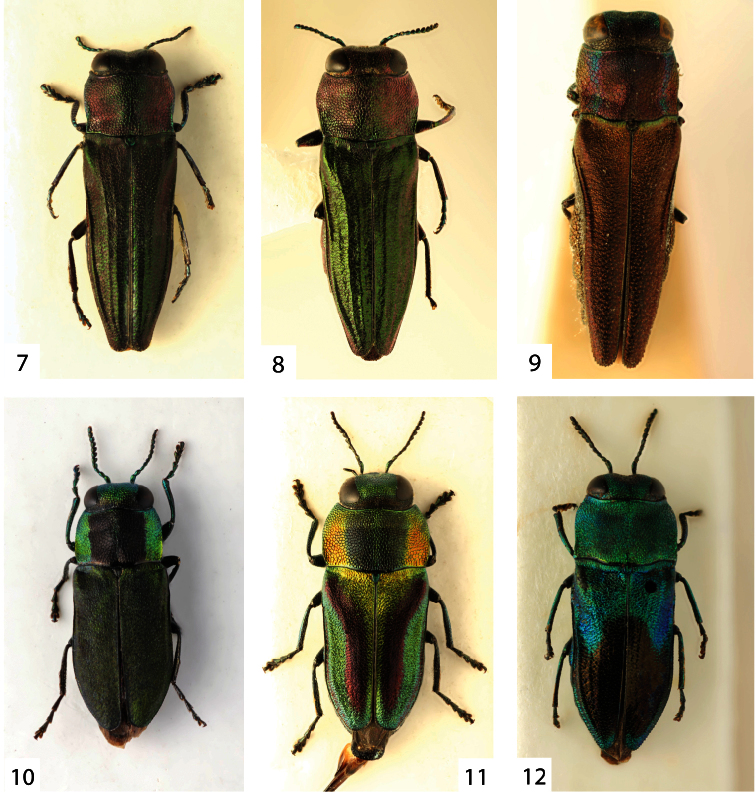
**7** *Agrilaxia (Costiptera) occidentalis* (Kerremans, 1900), male, 5.3 mm (Brasil, Caraça) **8** *Agrilaxia (Costiptera) costulipennis* (Cobos, 1972), male, 5.8 mm (Argentina, Entre Rios) **9** *Agrilaxia (Costiptera) modesta* (Kerremans, 1897), male, 5.3 mm (Brasil, Jatahy) **10** *Bilyaxia (Bilyaxia) cupriceps* (Fairmaire & Germain, 1858), male, 4.7 mm (Chile, La Unión) **11** *Bilyaxia (Bilyaxia) obscurata* (Reed, 1873), male, 5.3 mm (Chile, Talca) **12** *Bilyaxia (Paraguayetta) mariae* (Cobos, 1956), female, 7.0 mm (holotype of *Anthaxia jacobi* Obenberger, 1958).

**Figures 13–18.**
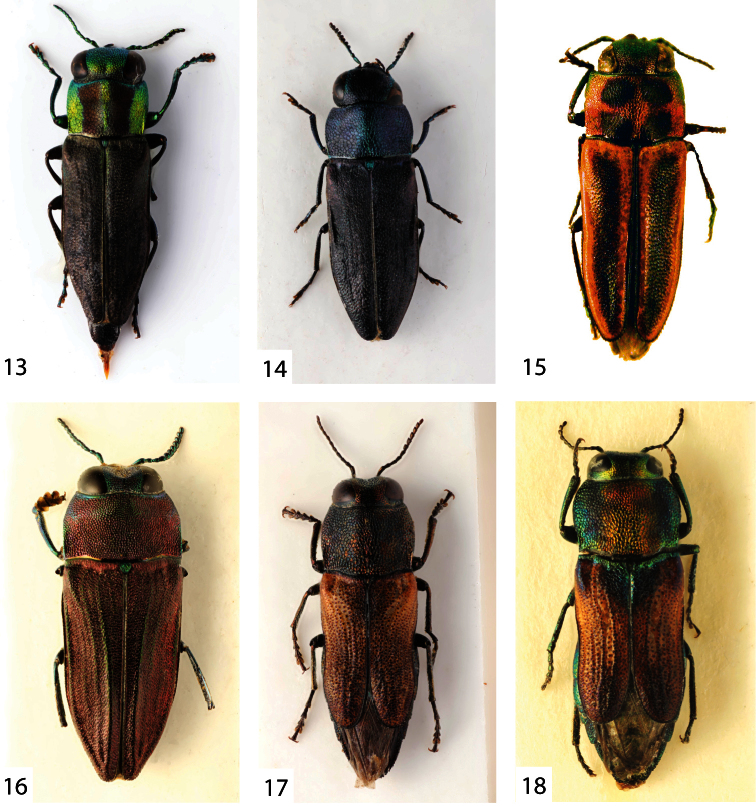
**13** *Bilyaxia (Tomasia) maculicollis* (Kerremans, 1887), female, 4.6 mm (Argentina, Entre Rios) **14** *Bilyaxia (Tomasia) lata* (Kerremans, 1903), male, 5.0 mm (Brasil, Rio Grande do Sul) **15** *Bilyaxia (Tomasia) cinctipennis* (Kerremans, 1913), male holotype, 7.0 mm **16** *Brasilaxia costifera* (Obenberger, 1913), male, 6.3 mm (Brasil, Curityba) **17** *Paracuris bimaculata bimaculata* (Gory, 1841), male, 7.1 mm (Argentina, Catamarca) **18** the same, female, 7.2 mm (Brasil, Bermejo).

**Figures 19–24.**
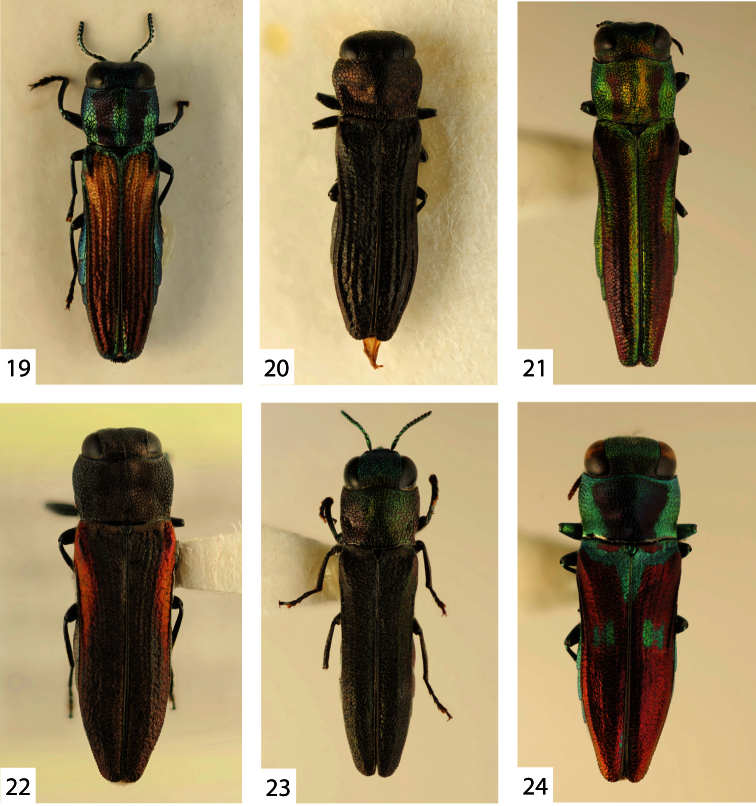
**19** *Agrilaxia (Agrilaxia) brunneipennis brunneipennis* (Kerremans, 1900), male, 4.9 mm (Argentina, Cordoba) **20** *Agrilaxia (Agrilaxia) coriacea* (Kerremans, 1887), female, 4.1 mm (Argentina, Misiones) **21** *Agrilaxia (Agrilaxia) kerremansi* (Théry, 1909), female, 6.1 mm (Brasil, Jatahy) **22** *Agrilaxia (Agrilaxia) montana* Bílý & Westcott, 2005, female paratype, 6.7 mm (Mexico, Sta Catarina del Monte) **23** *Agrilaxia (Agrilaxia) funebris* (Kerremans, 1900), male, 4.7 mm (Argentina, Entre Rios) **24** *Agrilaxia (Agrilaxia) alterna* (Kerremans, 1900), female, 5.5 mm (Brasil, Nova Teutonia).

**Figures 25–30.**
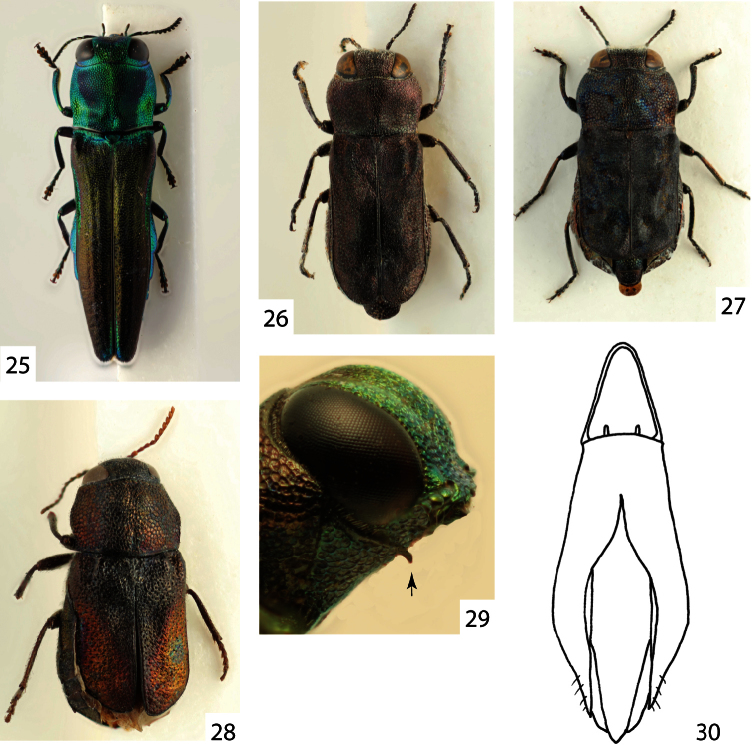
**25** *Agrilaxia (Agrilaxia) claudei* (Cobos, 1972), female, 7.4 mm (French Guayane, Kaw) **26** *Anilaroides brasiliensis* (Kerremans, 1897), male lectotype (Brasil, Bahia) **27** *Tetragonoschema (Tetragonoschema) undatum* (Steinheil, 1874), female, 4.0 mm (Paraguay, Loma Plata) **28** *Tetragonoschema (Patagoschema) patagonicum* (Obenberger, 1922), female lectotype, 4.6 mm **29** *Agrilaxia (Agrilaxia) decolorata* (Kerremans, 1899), male, prosternum **30** *Cobosina willineri* (Cobos, 1972), aedeagus.

**Figures 31–34.**
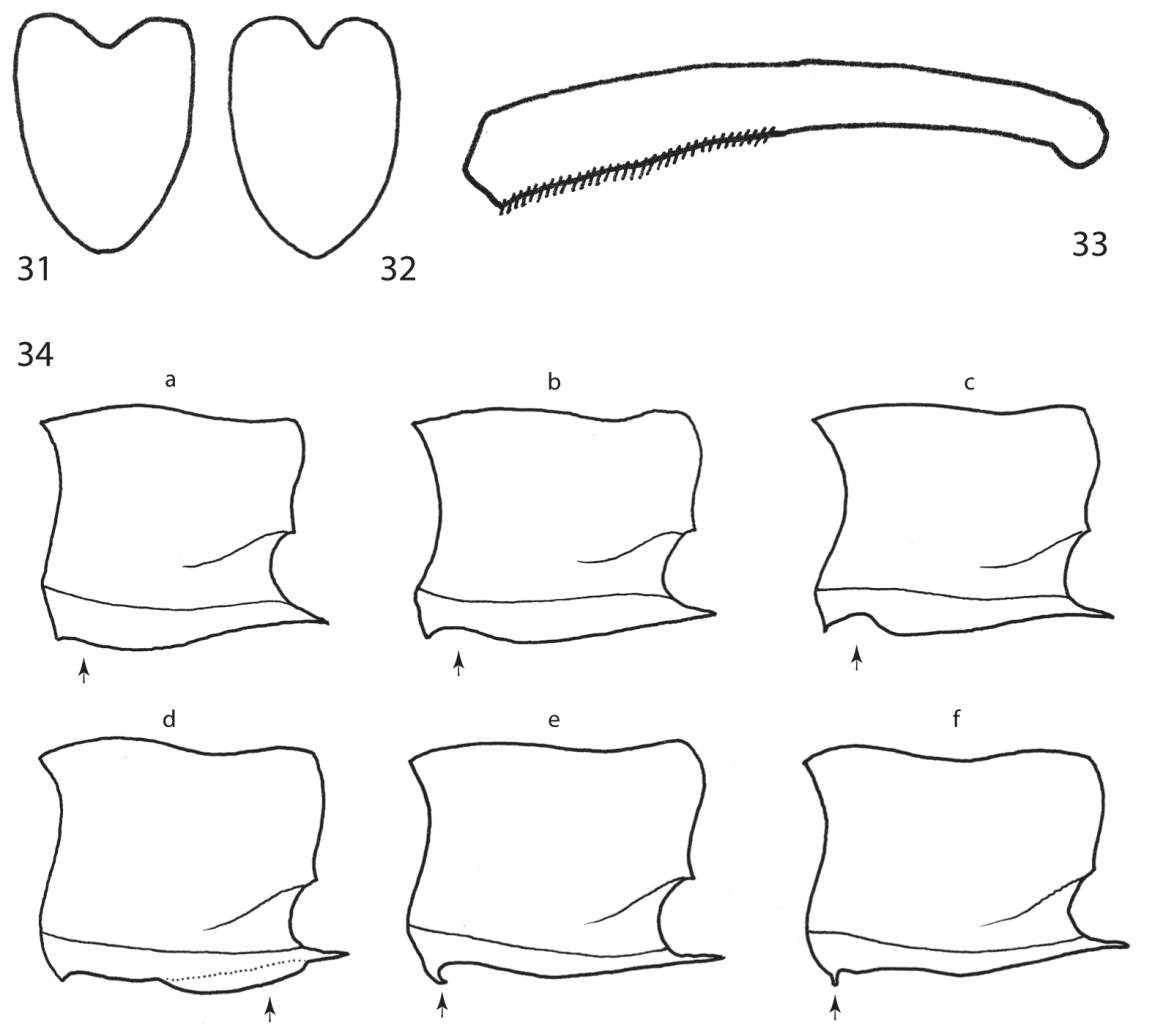
**31** *Charlesina mrazi* (Obenberger, 1932), scutellum **32** the same, *Anthaxita peruviana*, sp. n. **33** *Agrilaxia (Agrilaxia) claudei* (Cobos, 1972), right protibia **34** *Agrilaxia* spp., anterior margin of prosternum (lateral view): (**a**) flat to weakly convex, (**b**) weakly grooved, (**c**) deeply grooved, (**d**) with sternal, longitudinal carina, (**e**) rolled up anterior margin, (**f**) peg-like spine.

#### Anthaxita gen. n.

urn:lsid:zoobank.org:act:D7D526F5-4A92-405A-B6F9-84569DA5FE84

http://species-id.net/wiki/Anthaxita

Figs 1 , 32

##### Type-species.

*Anthaxita peruviana* gen. n., sp. n. by present designation.

##### Description.

Medium-sized (5.4 mm), dark, with silky lustre, *Anthaxia*-like, subparallel (Fig. 1); dorsal surface asetose, ventral surface with sparse, white, recumbent pubescence.

Head large, as wide as anterior pronotal margin; frons flat, vertex almost 3 times as wide as width of eye; antennae very short reaching anterior third of pronotal margins when laid alongside; clypeus short, transverse, anterior margin weakly emarginate.

Pronotum regularly convex, lateroposterior depressions and “agriloid” carina missing; lateral pronotal carina short, reaching posterior third of pronotal margin; posterior pronotal margin (covered by elytral base) serrate; posterior pronotal angles obtuse-angled; prescutellar pit and basal tubercles missing; pronotal sculpture very fine, homogeneous, consisting of very fine polygonal cells. Scutellum cordiform, 1.5 times as long as wide (Fig. 32), resembling the scutellum of the click-beetles of the subfamily Cardiophorinae.

Elytra about twice as long as wide, subparallel at anterior two thirds, regularly tapering at posterior third; elytral apices narrowly rounded, very finely serrate; elytral epipleura very narrow, narrowing posteriorly, reaching posterior fourth of elytral margin; subhumeral carina well defined; humeral swellings small; basal, elytral, transverse depression of elytra deep, narrow, reaching scutellum but interrupted by small tubercle near humeri; elytral sculpture very fine, homogeneous.

Ventral surface lustrous, metepisterna with patch of cream-white pubescence; prosternum weakly convex, anterior margin straight; prosternal process flat, slightly tapering posteriorly, not widened behind procoxae. Suture between abdominal ventrites 1–2 missing; anal tergite concave, posterior margin obtusely rounded, not serrate; anal ventrite slightly convex with obtusely rounded and finely serrate posterior margin. Legs short, stout, tarsi much shorter then tibiae. Tarsal claws strongly curved, robust, slightly enlarged at base.

##### Etymology.

The genus name *Anthaxita* gen. n. (feminine) indicates the strong similarity to the genus *Anthaxia*.

##### Differential diagnosis.

Except for the characters mentioned in the key, the genus *Anthaxita* gen. n. differs from other Neotropical genera of Anthaxiina by the combination of the following characters: pubescence of ventral surface, very wide vertex, very fine sculpture of pronotum and elytra, serrate posterior margin of pronotum, shortened elytral epipleura, missing suture between abdominal ventrites 1–2, short lateral pronotal carina, posteriorly acuminate prosternal process, concave anal tergite (pygidium), narrowly attenuate elytral apex and by the absence of the “agriloid” carina, lateroposterior pronotal depressions, and prescutellar pit. The male is unknown.

##### Distribution.

Peru.

##### Note.

Among all Neotropical Anthaxiini,the genus *Anthaxita* gen. n. is most similar to the Holoarctic species of *Anthaxia*, particularly to the subgenus *Melanthaxia* Rikhter, 1949.

#### Anthaxita peruviana gen. n., sp. n.

urn:lsid:zoobank.org:act:747B17D7-F025-4D40-AA44-3936D628F55D

http://species-id.net/wiki/Anthaxita_peruviana

Figs 1 , 32

##### Type locality.

Peru, Cusco.

##### Type specimen studied.

Holotype (female, NMPC): “Pérou (Cusco) Gay 59–49”.

##### Diagnosis.

Medium-sized (5.4 mm), black-bronze, matt with silky lustre (Fig. 1); clypeus and anterior portion of frons with weak, violet lustre; ventral surface lustrous, black-violet; entire dorsal surface asetose, ventral surface with rather long, sparse, recumbent white pubescence, metepisterna covered with cream-white tomentum.

##### Description of female holotype.

Head wide, large, as wide as width of anterior pronotal margin; clypeus twice as wide as long, separated from frons by shallow, transverse impression, anterior margin widely, shallowly emarginate; frons flat with two shallow, rounded depressions above antennal insertions; vertex flat, 2.8 times as wide as width of eye; eyes relatively small, elliptical, not projecting beyond outline of head; antennae very short, finely serrate from fourth antennomere; scape pyriform, slightly flattened, 3.5 times as long as wide; pedicel suboval, 1.5 times as long as wide; third antennomere weakly triangular, 1.5 times as long as wide; antennomere 4 sharply triangular, 1.3 times as long as wide; antennomeres 5–10 trapezoidal, 1.3 times as wide as long, terminal antennomere rhomboid, somewhat longer than wide; sculpture of head consisting of small, fine, very dense polygonal cells with microsculptured bottoms.

Pronotum almost regularly convex, 2.2 times as wide as long, slightly flattened at posterior angles; anterior margin deeply biarcuate, medial lobe large, strongly projecting forward, posterior margin nearly straight; lateral margins regularly, widely rounded, posterior angles obtuse-angled; prescutellar pit and “agriloid” carina missing; sculpture consisting of very fine, small, polygonal cells, nearly indistinct on pronotal disc. Scutellum (Fig. 31) 1.3 times as long as wide, cordiform, flat, lustrous, microsculptured.

Elytra 2.2 times as long as wide, regularly convex, subparallel, regularly tapering at posterior third; humeral swellings slightly projecting beyond outline of elytra; each elytron narrowly rounded apically, with quite indistinct serrations; subhumeral carina well defined, flat, reaching apical third of lateral margins; elytral epipleura narrow, not very well defined, becoming narrower posteriorly, reaching posterior fifth of elytral margins; elytral sculpture very fine, consisting of tiny, transverse, zig-zag rugae which are much denser on humeral portion of elytra than that on disc.

Ventral surface lustrous, finely ocellate, anal ventrite widely triangular, apically subtruncate, finely serrate laterally. Legs short, stout, all tibiae straight, unmodified; tarsi short, tarsomeres 1–4 rather wide with well defined, ventral, adhesive pads; all tarsi much shorter than corresponding tibiae. Tarsal claws robust, strongly hook-shaped, slightly enlarged at base.

Sexual dimorphism. Male unknown.

Measurements. Length: 5.4 mm; width: 2.0 mm.

##### Etymology.

*Anthaxita peruviana* sp. n. is named after the country of origin (Peru).

##### Differential diagnosis.

All differential characters are given in the description of the genus.

##### Distribution.

Peru.

##### Note.

The holotype was originally pinned, so the right elytron and corresponding portion of the abdomen are somewhat damaged (see Fig. 1).

#### Charlesina gen. n.

urn:lsid:zoobank.org:act:EB52AC1B-1536-44D7-8ED1-A38250C9CDB0

http://species-id.net/wiki/Charlesina

Figs 2 , 31

##### Type species.

*Agrilaxia mrazi mrazi* Obenberger, 1932 by present designation.

##### Description.

Medium-sized (5.5 mm), black, elongate, matt, entirely asetose; lateral margins of elytra with green tinge.

Head as wide as anterior pronotal margin; clypeus narrow, trapezoidal, anterior margin deeply emarginate; frons widely grooved; vertex flat, twice as wide as width of eye; eyes broad elliptical, not projecting beyond outline of head; antennae short and robust, scarcely reaching midlength of lateral pronotal margins when laid alongside; scape broad pyriform, twice as long as wide; pedicel subcylindrical, 1.4 times as long as wide; third antennomere short, 1.2 times as long as wide; antennomeres 4-10 short, trapezoidal, nearly twice as wide as long; terminal antennomere rhomboid, 1.5 times as wide as long; sculpture of head consisting of fine, simple, sparse punctures on frons and weakly defined, dense cells on vertex.

Pronotum twice as wide as long, with wide, deep lateroposterior depressions, without distinct sculpture; lateral margins weakly S-shaped, posterior angles right angled; “agriloid” carina well defined, rather sharp, as long as lateral pronotal carina; basal tubercles weakly defined, prescutellar pit small, deep, well defined. Scutellum small, cordiform (Fig. 31), flat, twice as long as wide, resembling the scutellum of the click-beetles of the subfamily Cardiophorinae.

Elytra elongate, 2.3 times as long as wide, widely sphenoidal with shallow, longitudinal depression at posterior fourth; humeral swellings small but well defined, basal, transverse depression deep, reaching scutellum; elytral epipleura strongly reduced, very narrow, not reaching apex of elytra; subhumeral carina well defined, reaching apical portion of elytra; elytral sculpture consisting of fine microsculpture and very small, lustrous grains. Anal ventrite with fine but sharp, lateral serrations; all tarsi distinctly widened; tarsal claws simple, strongly hook-shaped.

Aedeagus (Figs 130, 131 in Cobos 1972) long, slender, spindle-shaped, setiferous part of parameres very short, median lobe pointed apically.

##### Etymology.

The new genus *Charlesina* gen. n. (feminine) is dedicated to my friend and colleague, Charles Bellamy (Sacramento, California), one of the best world specialists in the taxonomy of Buprestidae, with my warm thanks for his life long cooperation.

##### Differential diagnosis.

The monotypic genus *Charlesina* gen. n. strongly differs from other Neotropical Anthaxiina except for the genus *Sanchezia* gen. n. Both genera share some general characters like black, flattened body without distinct sculpture of dorsal surface, widened, robust antennae and tarsi, well defined lateroposterior pronotal depressions and “agriloid” carina. The genus *Charlesina* gen. n. strongly differs from the genus *Sanchezia* gen. n. by the deeply grooved frons, well defined humeral swellings and basal, transverse, elytral depression, by the narrow, reduced elytral epipleura, long, well defined subhumeral carina and by the entirely asetose body.

##### Species included.

*Charlesina mrazi* (Obenberger, 1932).

##### Distribution.

Argentina, Brasil.

##### Note.

Also this genus can be included among the Lycid immitating Buprestids (see *Sanchezia* gen. n. below).

#### Charlesina mrazi mrazi

(Obenberger, 1932) comb. n.

http://species-id.net/wiki/Charlesina_mrazi_mrazi

Figs 2 , 31 ; Figs 130, 131 in Cobos 1972

Agrilaxia mrazi Obenberger, 1932: 144. Type locality: Brasil.
Agrilaxia mrazi : Blackwelder, 1944: 314 (checklist).
Anthaxia (Agrilaxia) mrazi Cobos, 1972: 203 (revision, key).
Anthaxia (Cylindrophora) mrazi : Bílý, 1997: 29, 94 (catalogue).
Cylindrophora mrazi : Bellamy, 2008: 1298 (catalogue).

##### Type specimen studied.

Syntype (♂, NMPC): “Brazil, Rio, Itatiaya[h]//Typus[p]//Mus. Nat. Pragae Inv. 22638[p]// *Agrilaxia mrazi* m. Type, Det. D^r.^ Obenberger[h+p]”; since the number of syntypes is unknown and to avoid any confussion in the future I designate hereby this specimen as the lectotype.

##### Further specimen studied.

*Anthaxia (Agrilaxia) mrazi* ssp. *cyaneobscura* Cobos, 1972: holotype by monotypy (♂, MNCN): “Vila Oliva, Rio Grande do Sul, P. Buck[p]// *Anthaxia (Agrilaxia) mrazi cyaneobscura* ssp. n.[h] Dr. A. Cobod det.[p] ”.

Aedeagus (Figs 130, 131 in Cobos 1972) spindle-shaped, setiferous portion of parameres very short, both parameres and median lobe sharply pointed apically.

Length: 5.8 mm; width: 1.7 mm.

##### Distribution.

Brasil (Rio de Janeiro, Rio Grande do Sul).

##### Note.

The locality data given in the description (Obenberger, 1932) slightly differ from those on the syntype (see above); although the number of syntypes is unknown, there is no doubt that the species was described from this specimen which is hereby designated as the lectotype (see above).

Cobos (1972), although treated this species in the genus *Anthaxia* subgen. *Agrilaxia*, noticed that this species should be probably treated in the genus *Cylindrophora*. This opinion was accepted by Bílý (1997) and Bellamy (2008). After the re-definition of the monotypic genus *Cylindrophora* (*Cylindrophora maulica*) Molina, 1782)) and its transfer to the subtribe Curidina (Bílý, 2004), *Agrilaxia mrazi* (together with several other species of the former *Cylindrophora*) was formally attributed to the genus *Bilyaxia*. This act was overlooked by Bellamy (2008) who still treated *Charlesina mrazi* in the genus *Cylindrophora* sensu Cobos, 1956 and 1972.

Cobos (1972) described *Anthaxia (Agrilaxia) mrazi* ssp. *cyaneobscura* Cobos, 1972 on a single male from south-eastern Brasil (Rio Grande do Sul). This subspecies differs from *Charlesina mrazi mrazi* only slightly by its colouration and by the more robust aedeagus (Figs 130, 131 in Cobos 1972). More specimens are needed to resolve the taxonomic status of this subspecies.

#### Cobosina gen. n.

urn:lsid:zoobank.org:act:CC52C856-8F7F-4DF8-8796-93B4206EAC98

http://species-id.net/wiki/Cobosina

Figs 3 , 30

##### Type species.

*Anthaxia (Cylindrophora) willineri* Cobos, 1972 by present designation.

##### Description.

Small (4.25 mm), subcylindrical, rather convex, subparallel, entirely asetose.

Head small, mouth parts small, only slightly visible, partly retracted into head; frontoclypeal portion of head rathed reduced, clypeus very short, widely transverse, with straight anterior margin; frons strongly convex; vertex about twice as wide as width of eye; eyes rather large, elliptical, not projecting beyond outline of head; antennae very short, reaching anterior third of pronotal margins when laid alongside; antennomeres 4–11 obtusely triangular to trapezoidal, wider than long.

Pronotum strongly convex, lateral margins without lateral carina; lateroposterior depressions almost indistinct, “agriloid” carina very short, flat; prosternum with very narrow, transverse groove just behind anterior margin; prosternal proceess flat, short, nearly triangular, not widened behind procoxae. Scutellum small, longer than wide.

Elytra moderately convex, with small humeral swellings; basal transverse depression wide, nearly reaching scutellum; elytral apices separately, widely rounded, with very fine apical serrations; posthumeral carina short, flat, weakly defined, reaching only level of metacoxae; elytral epipleura very narrow, scarcely visible, reaching posterior fourth of elytra.

Anal ventrite wider than long, slightly truncate apically, with very fine, lateral serrations; legs simple, male metatibiae not modified; tarsal claws thin, simple, as long as half of terminal tarsomere; aedeagus (Fig. 30) very short, widely spindle-shaped, with the maximum width at the posterior third of the parameres, which are sharply pointed; medial lobe wide, pointed apically, without lateral serrations.

##### Etymology.

The genus *Cobosina* gen. n. (feminine) is named in honour of my late colleague and well-known specialist in the taxonomy of the Neotropical Buprestidae, Prof Antonio Cobos Sánchez (Almería, Spain).

##### Differential diagnosis.

Although the genus *Cobosina* gen. n. resembles very much some European species of the genus *Anthaxia* (see below) it shares some principal characters with the Neotropical Anthaxiini: completely asetose body (only a few species of *Anthaxia* from south-eastern Asia are entirely asetose), prolonged scutellum which is longer than wide, narrow and shortened elytral epipleura, “agriloid” carina, posthumeral carina (the latter two rather reduced), obtusely rounded elytral apex and simple, spindle-shaped aedeagus.

The genus *Cobosina* gen. n. differs from other genera of the Neotropical Anthaxiina by the small, non-prognathous mouth parts which are partly retracted into the head, reduced, very short clypeus which is not separate from the frons (most of the Neotropical Anthaxiina are more or less prognathous), by the asetose protibiae (very often with the brush-like, stiff, pale setae on inner margin of protibiae in other Anthaxiina, namely *Agrilaxia*– Fig. 33) and last but not least by the clearly *Anthaxia*-like appearance.

##### Distribution.

Argentina, Brasil.

#### Cobosina willineri

(Cobos, 1972) comb. n.

http://species-id.net/wiki/Cobosina_willineri

Figs 3 , 30

Anthaxia (Cylindrophora) willineri Cobos, 1972: 224. Type locality: Argentina, Valle Fértil, Prov. San Juan.
Anthaxia (Bilyaxia) willineri : Bílý 1997: 39, 128 (catalogue); Bellamy 2008: 1497 (catalogue).

##### Type specimen studied.

Holotype by monotypy (♀, MNCN): “[Argentina]Valle Fértil, San Juan, Rvdo. P. G. Williner coll., 5-6.xi.1970[p]”.

##### Further specimen studied.

“Brasil, Catamarca, 6 km N Belém, 1240 m[h]” (1♂, NMPC).

##### Note.

The species was formally attributed tothe genus *Bilyaxia* by Bílý (2004). Already in the original description (Cobos, 1972) noted that this species is an extraordinary and isolated element of the Neotropical Anthaxiini being very similar to the European species of the *Anthaxia (Anthaxia) funerula* Illiger, 1803 species-group. After having studied the holotype and further, male specimen of this species from NMPC I found that *Anthaxia (Bilyaxia) willineri* ought to be removed from *Bilyaxia*, and a new genus should be proposed for this species.

I failed to find any difference between the sexes and also no significant variability since only two specimens were available for study.

Length: 4.2–4.4 mm; width: 1.4–1.5 mm.

##### Distribution.

Argentina (Prov. San Juan); Brasil (Prov.Catamarca), new country record.

#### Marikia gen. n.

urn:lsid:zoobank.org:act:0F360354-DDAE-4677-ACAA-6654DA61B4A6

http://species-id.net/wiki/Marikia

Fig. 4 ; Figs 76, 77 in Cobos 1956

##### Type species.

*Anthaxia (Cylindrophora) descarpentriesi* Cobos, 1956 by present designation.

##### Description.

Medium-sized (4.0–5.5 mm), flat, black, lustrous, entirely asetose.

Head as wide as anterior pronotal margin; clypeus broadly trapezoidal, anterior margin finely emarginate; frons flat to weakly depressed, vertex flat, 2.5 times as wide as width of eye; eyes reniform to elliptical, not projecting beyond outline of head; antennae filiform, slightly overlapping midlength of lateral pronotal margins when laid alongside; pedicel as wide as scape, 1.5 times as long as wide; antennomeres 4–10 sharply triangular to trapezoidal, terminal antennomere prolonged, 2.5 times as long as wide.

Pronotum moderately convex, 2.4 times as wide as long, posterior angles obtuse-angled; lateroposterior depressions small, shallow, situated at posterior corners of pronotum; “agriloid” carina sharp, reaching midlength of lateral margins, very close to the lateral pronotal carina; pronotal sculpture consisting of very fine, almost indistinct, transversely widened cells and rugae; basal tubercles missing, prescutellar pit wide but shallow. Scutellum small, triangular, weakly depressed, microsculptured, slightly longer than wide.

Elytra 2.2 times as long as wide, weakly convex, somewhat uneven, parallel-sided at anterior two thirds, nearly regularly tapering at posterior third; apices narrowly rounded, very slightly caudiform, without distinct serrations; humeral swellings well defined, transverse basal depression deep, wide, reaching scutellum, interrupted by lustrous tubercle near humeri; elytral epipleura narrowing posteriorly, reaching apical third of elytral margins; subhumeral carina indistinct; elytral sculpture consisting of simple punctures and short, irregular, transverse rugae; elytral suture strongly elevated in posterior half.

Suture between ventrites 1 and 2 missing; anal ventrite widely rounded, with fine lateral serrations, anal tergite truncate without distinct serrations. Prosternum flat, prosternal process slightly enlarged behind procoxae. Legs long, unmodified, tarsi somewhat widened. Tarsal claws simple, slender, moderately curved.

Aedeagus (Figs 76, 77 in Cobos 1956) short, widely spindle-shaped, median lobe pointed apically.

##### Etymology.

The genus *Marikia* gen. n. (feminine) is named in the honour of my life long friend and colleague Mark (Marik) G. Volkovitsh (St. Petersburg, Russia) with many thanks for his help, cooperation and hospitality during my stays in St. Petersburg.

##### Differential diagnosis.

The genus *Marikia* gen. n. somewhat resembles by its dark colouration, flattened body and narrowly acuminate elytra some small species of the tribe Melanophilini Bedel, 1921. Among the Neotropical Anthaxiina it is most similar to the genus *Anthaxita* gen. n. from which it differs (except for the characters given in the key) by the lustrous and flat, entirely asetose body, narrow clypeus, longer and slender antennae, indistinct pronotal sculpture, long, sharp “agriloid” carina, triangular scutellum, posteriorly widened prosternal process, uneven and somewhat caudiform elytra, strongly elevated elytral suture and by the rudimental subhumeral carina.

##### Distribution.

Ecuador.

#### Marikia descarpentriesi

(Cobos, 1956) comb. n.

http://species-id.net/wiki/Marikia_descarpentriesi

Fig. 4 ; Figs 76, 77 in Cobos 1956

Anthaxia (Cylindrophora) descarpentriesi Cobos, 1956: 154. Type locality: Ecuador, Quito.
Anthaxia (Cylindrophora) descarpentriesi : Cobos 1990: 53 (note, new records);
Anthaxia (Bilyaxia) descarpentriesi : Bílý 1997: 20, 65 (catalogue);
Bilyaxia descarpentriesi : Bellamy 2008: 1496 (catalogue).

##### Type specimens studied.

Holotype (male, MNHN): “Quito, Equateur, Benoit, i.1932[h]”; allotype (female, MNHN): “Puembo, Equateur, Benoit, 20.ii.1931[h]”.

##### Further specimens studied.

**ECUADOR:** “Pichincha-Quito, 23.vi.1985, Gustavo Morejón[p]” (1 male, 1 female, MNCN); “Pichincha, Alluriquin, 6.v.1948, F. Grazo leg.[h]” (1 male, NMPC).

No difference between both sexes were observed and also no variability in the colouration apparent.

Aedeagus (Figs 76, 77 in Cobos 1956) very short, widely spindle-shaped, median lobe simply pointed apically.

Length: 4.0–5.5 mm; width: 1.7–2.0 mm.

##### Distribution.

Ecuador.

##### Note.

Terminal antennomere unusually long, 2.5 times longer than wide which is rather strange character within Anthaxiini.

#### Sanchezia gen. n.

urn:lsid:zoobank.org:act:1E18DD6B-BB25-4E5A-B1AC-4224EA031247

http://species-id.net/wiki/Sanchezia

Fig. 5 ; Fig. 2 in Bílý 1996

##### Type species.

*Anthaxia (Cylindrophora) bucki* Cobos, 1956 by present designation.

##### Description.

Rather large (5.7–7.0 mm), flat, Lycid-like, matt; antennae and legs robust; dorsal surface asetose, frons and ventral surface with sparse but rather long, white pubescence, prosternum with lanuginose pubescence.

Head wider than anterior pronotal margin, eyes large, elliptical, projecting beyond outline of head; clypeus short, wide, anterior margin straight; frons flat with small, rounded cells with tiny central grains (central portion of frons with rather sparse cells), vertex flat, 1.6 times as wide as width of eye, with fine, transverse rugae; antennae reaching posterior fourth of lateral, pronotal margins when laid alongside; pedicel triangular, antennomeres 3–10 widely triangular to trapezoidal, much wider than long.

Pronotum tapering anteriorly, 2.2 times as wide as long, with fine, medial, longitudinal depression and large, deep lateroposterior depressions reaching anterior fourth of pronotum; “agriloid” carina well defined, long, reaching beyond midlength of lateral margins; prescutellar pit missing; pronotal sculpture consisting of very fine, rather indistinct and transversely widened, polygonal cells without central grains. Scutellum very small, triangular, 1.5 times as long as wide.

Elytra flat, 1.8–1.9 times as long as wide, narrowly, separately rounded apically; humeral swellings small, not projecting beyond outline of elytra, basal, transverse depression missing; elytral epipleura wide, enlarged posteriorly, reaching elytral suture; subhumeral carina missing; elytral sculpture consisting of fine microsculpture and tiny, sparse, lustrous grains.

Ventral surface lustrous, abdominal ventrites with fine horse-shoe-shaped punctures; prosternum weakly convex, densely, transversely rugate, prosternal process enlarged beyond procoxae; anal ventrite truncate, sharply serrate, anal tergite spatulate, unarmed. Legs robust, protibiae slightly curved with inner, preapical, brush-like row of dense, pale bristles (denser in males – like in Fig. 33); both meso- and metatibiae finely, obtusely serrate on inner margin; tarsal claws thin, hook-shaped.

Aedeagus (Fig. 2 in Bílý 1996) subparallel, strongly sclerotised, parameres sharply pointed apically; median lobe apically obtusely pointed.

##### Etymology.

The genus *Sanchezia* gen. n. (feminine) is named in the honour of my late colleague Prof Antonio Cobos Sánchez (Almería, Spain), the well-known specialist in the taxonomy of the Neotropical Buprestidae.

##### Differential diagnosis.

The genus *Sanchezia* gen. n. is quite unmistakable, characteristic genus which is somewhat similar to the genus *Charlesina* gen. n. (see above). It differs from other taxa of the Neotropical Anthaxiini by the rather non-Buprestid appearence, very fine, nearly velvet texture of dorsal surface, robust antennae with antennomere 3 triangularly enlarged, very wide elytral epipleura and other characters mentioned in the key.

##### Distribution.

Argentina, Brasil.

#### Sanchezia bucki

(Cobos, 1956) comb. n.

http://species-id.net/wiki/Sanchezia_bucki

Fig. 5 ; Fig. 2 in Bílý 1996

Anthaxia (Cylindrophora) bucki Cobos, 1956: 162. Type locality: Brasil, Porto Alegre.
Anthaxia (Bilyaxia) bucki : Bílý 1997: 16, 54 (catalogue);
Bilyaxia bucki : Bílý 2004: 8 (taxonomy, synonymy); Bellamy 2008: 1495 (catalogue);
Anthaxia (Cylindrophora) kafkai Bílý, 1996: 28. Type locality: Argentina, Entre Rios, Pronunciamiento.
Anthaxia (Cylindrophora) kafkai : Bílý 1997: 26, 83 (catalogue); 1999: 236 (catalogue); 2004: 8 (synonym of *bucki*); Bellamy 2008: 1495 (catalogue, as syn. of *bucki*).

##### Type specimens studied.

***Anthaxia bucki***: Holotype (female, MNCN): “Porto Alegre[h]// *Anthaxia (Cylindrophora) bucki* sp. n.[h] Dr. A. Cobos det.[p]”; ***Anthaxia kafkai*:** Holotype (male, NMPC): “Argentina, Entre Rios, Pronunciamiento, ix.1992[p]”.

##### Further specimen studied.

**BRASIL:** “RS [main road] Canela, 22.ix.1985, leg. G. Scherer[p]” (1 female, NMPC).

No difference was observed between the sexes. It was impossible to study the variability of this species since only one specimen was found in the collections, except for the type specimens.

Length: 5.7–7.0 mm; width: 2.2–2.4 mm.

##### Distribution.

Argentina, Brasil.

##### Note.

Also Cobos (1956) stressed in his description the strange appearence of this species and attributed it to *Anthaxia* with strong doubts. *Sanchezia bucki* and *Charlesina mrazi* are further Neotropical species of Buprestidae mimicking Lycidae. As far as I know, there are only some species of the genus *Agrilus* (*Agrilus dilaticornis* Kerremans, 1897 species-group) and Chilean species *Philandia valdiviana* (Philippi & Philippi, 1860) mimicking Lycid beetles.

#### Agrilaxia

Kerremans, 1903

http://species-id.net/wiki/Agrilaxia

Figs 6 , 19 –25

##### Type species.

*Anthaxia flavimana* Gory, 1841 (subsequent designation: Chamberlin, 1926).

The genus *Agrilaxia* was described by Kerremans (1903) and 19 mostly Neotropical species were included without designation of a type species. *Agrilaxia flavimana* Gory, 1841 was subsequently designated by Chamberlein (1926) as the type species of the genus. Later on, the genus *Agrilaxia* was synonymized by Théry (1930) with *Anthaxia* Eschscholtz, 1829, ressurected again as a valid genus by Obenberger (1930), then finally treated as a subgenus of *Anthaxia* by Cobos (1972); this concept was followed also by Bílý (1984, 1993). Bílý & Bellamy (1999) upgraded *Agrilaxia* again to the generic level which was followed by Bílý (2000, 2004) and Bellamy (2008).

The genus was revised by Cobos (1972) (as a subgenus of *Anthaxia*) who described the most of the Neotropical species. Subsequently several species were described also by Bílý (1984, 1985, 1993), Bílý & Westcott (2005), Cobos (1986) and by Bílý & Brûlé (in press). The morphology and the characters specific to the genus *Agrilaxia* were discussed by Cobos (1972) and Bílý & Brûlé (in press).

Even after separation of some species as independent genera (see above), the genus remains still rather heterogeneous. I propose that first, the small and rather homogeneous group of species with short, green or violet, wedge shaped body should be split as the separate subgenus *Costiptera* subgen. nov (see below).

#### Agrilaxia (Costiptera) subgen. n.

Figs 7–9

##### Type species.

*Anthaxia occidentalis* Kerremans, 1900 by present designation.

##### Diagnosis.

Body short, wedge shaped, ventral surface with short but distinct, white pubescence, metepisterna and metacoxae often with a patch of white tomentum; dorsal surface green, golden green or violet-green, sometimes pronotum black or violet with two longitudinal, green stripes.

Head large, often wider than anterior pronotal margin, weakly prognathous; clypeus short, transverse, anterior margin straight; frons widely, deeply grooved; vertex shallowly depressed, about twice as wide as width of eye; antennae short, rather robust, reaching midlength of lateral pronotal margins when laid alongside; sculpture of frons consisting of small, dense, polygonal cells with small central grains.

Pronotum relatively narrow, convex, 1.8–2.0 times as wide as long, with well defined lateroposterior depressions situated near posterior angles; “agriloid” carina well defined, short, close to lateral carina; sculpture homogeneous, consisting of small, almost regular, polygonal cells, with or without central grains; prescutellar pit shallow, basal tubercles missing or very weakly defined. Scutellum small, flat, usually longer than wide.

Elytra convex, strongly wedge shaped, about 2.5 times as long as wide, large parts of abdominal ventrites well visible from above; elytra smooth or with 2–3 longitudinal costae; elytral epipleura narrow, nearly reaching elytral apex; basal, transverse depression deep, reaching scutellum, subhumeral carina well defined, long.

Prosternum flat or weakly convex, anterior margin sometimes weakly, narrowly elevated; both anal ventrite and sternite finely serrate.

##### Etymology.

The name of the new subgenus *Costiptera* subgen. n. (feminine) is composed of the Latin substantive “*costa*” (carina, rib) and Greek substantive “*pteron*” (wing) to stress one of the main characters of the subgenus.

##### Differential diagnosis.

The subgenus *Costiptera* subgen. n. differs from the nominate subgenus particularly by shorter, wedge shaped elytra (usually 2.5 times as long as wide), pubescence of the ventral surface and by the set of characters given above and in the key.

##### Species included.

*Agrilaxia (Costiptera) ambigua* (Cobos, 1972), *Agrilaxia (Costiptera) clara* (Kerremans, 1899), *Agrilaxia (Costiptera) costulipennis* (Cobos, 1972), *Agrilaxia (Costiptera) interposita* (Cobos, 1972), *Agrilaxia (Costiptera) modesta* (Kerremans, 1897) and *Agrilaxia (Costiptera) occidentalis* (Kerremans, 1900).

##### Note.

Two species groups can be separated within the subgenus. *Agrilaxia (Costiptera) modesta* (Kerremans, 1897) species-group which is characterised by the smooth elytra, well defined central grains in the fine frontal and pronotal ocellation, shorter, pentagonal scutellum which is as wide as long and by the shorter subhumeral carina reaching only midlength of elytral margins and by the anterior margin of the prosternum bearing a peg-like spine (like in Figs 29, 34f); only *Agrilaxia (Costiptera) modesta* can be included into this species-group. For the remaining species, the *Agrilaxia (Costiptera) occidentalis* (Kerremans, 1900) species-group is suggested which is characterised by the longitudinal elytral costae, frontal and pronotal sculpture without central grains, prolonged scutellum which is 1.5 times longer than wide and by the long, well defined subhumeral carina reaching the elytral apex.

#### Agrilaxia (Agrilaxia)

Kerremans, 1903

Figs 6 , 19 –25

##### Type species.

*Anthaxia flavimana* Gory, 1841 (subsequent designation: Chamberlin, 1926).

According to Bellamy (2008), the genus *Agrilaxia* comprises 88 species. After removing of 5 species from *Agrilaxia* to *Bilyaxia* (see below) and after transferring of 5 species into the subgenus *Costiptera* subgen. n., 78 species remain in the subgenus *Agrilaxia* s. str. Quite naturally the subgenus will have to be split into several (or many) species-groups since several very different morphotypes (e.g. Figs 6, 19–25) can be found within the subgenus - some of them have been suggested by Bílý & Brûlé (in press).

##### Note.

The very special morphological character of the subgenus *Agrilaxia* is the shape of the prosternum (Figs 29, 34a–34f) which is normally flat or slightly convex. In the most species the prosternum is more or less transversely grooved just behind the anterior margin. The “grooving” of the anterior portion of prosternal plate and the relative elevation of the anterior margin of the prosternum which, in the extreme case, forms a sharp, transverse, rolled up ledge which is sometimes transformed into a peg-like, medial spine (Figs 29, 34f).

#### Bilyaxia

Hołyński, 1989

http://species-id.net/wiki/Bilyaxia

Figs 10 –15

##### Type species.

*Anthaxia cupriceps* Fairmaire & Germain, 1858 by original designation.

The genus was originally described by Hołyński (1989) as a subgenus of *Anthaxia* and it was upgraded to the genus level by Bílý (2004). According to Bellamy (2008) it contains 18 species but the division to several subgenera is necessary. Three species were transferred to the separate, monotypic genera (*Charlesina* gen. n., *Cobosina* gen. n. and *Marikia* gen. n. – see above), the remaining 15 species is assigned into three subgenera.

#### Bilyaxia (Bilyaxia) s. str.

Figs 10, 11

##### Type species.

*Anthaxia cupriceps* Fairmaire & Germain, 1858 by original designation.

##### Diagnosis.

Medium-sized (3.5–6.3 mm), subparallel, moderately convex, *Anthaxia*-like species.

Head large, very often somewhat wider than anterior pronotal margin; frons flat or slightly depressed, vertex 1.3–1.8 times as wide as width of eye; sculpture of head consisting of dense, oval or polygonal cells with or without central grains

Pronotum flattened, lateral margins subparallel or regularly rounded, posterior margin weakly biarcuate or almost straight, lateroposterior depressions usually well defined; “agriloid” carina short, almost parallel with lateral carina; prescutellar pit missing, basal tubercles small or missing. Scutellum small, subcordiform, usually 1.5 times as long as wide, flat.

Elytra moderately convex, subparallel, rarely weakly wedge shaped, 1.7–1.8 times as long as wide; humeral swellings well defined, elytral apices widely, obtusely rounded; basal, transverse depression rather deep, reaching or nearly reaching scutellum; elytral epipleura narrow, usually narrowing posteriorly, reaching elytral apex; subhumeral carina rather long, obsolete, not very elevated.

Ventral surface finely ocellate, prosternum flat or weakly convex; anal sternite obtusely rounded or truncate, serrate laterally. Legs moderately long, rather robust, tarsi distinctly widened, almost as long as tibiae.

##### Differential diagnosis.

The subgenus *Bilyaxia* differs from both newly described subgenera by flat, wide, *Anthaxia*-like body, flat or slightly depressed frons, shape of pronotum, obtusely rounded elytral apex, short elytra, distinctly widened tarsi and by the geographical distribution: all species are distributed west of the Andes mountains in Chile; only *Bilyaxia (Bilyaxia) auronotata* (Bílý, 1978) is distributed also in Argentina (Neuquén).

##### Note.

*Bilyaxia auronotata* was described from the Argentinian province Neuquén (Lago Lacar). The lake Lacar lies at the Chilean border at the lowest spot of this part of the Andean range; here is the only “gate” where the Buprestid species can pass through the Andes. *Bilyaxia auronotata* was rather frequently collected also in the opposite side in Chile (prov. Villarica).

The distribution of the genus *Bilyaxia* is similar in distribution to the genera *Conognatha* Eschscholtz, 1829 and *Dactylozodes* Chevrolat, 1838, the subgenera of these are also distributed on the opposite sides of the Andes.

##### Species included.

*Bilyaxia (Bilyaxia) auronotata* (Bílý, 1978), *Bilyaxia (Bilyaxia) concinna* (Mannerheim, 1837), *Bilyaxia (Bilyaxia) cupriceps* (Fairmaire & Germain, 1858), *Bilyaxia (Bilyaxia) cordillerae* (Obenberger, 1928), *Bilyaxia (Bilyaxia) obscurata* (Reed, 1873) and *Bilyaxia (Bilyaxia) rubricollis* (Moore, 1981).

#### Bilyaxia (Paraguayetta) subgen. n.

Fig. 12

##### Type species:

*Anthaxia (Cylindrophora) mariae* Cobos, 1956 by present designation.

##### Diagnosis.

Rather large (7.0 mm), robust, moderately convex, multicolorous: dorsal surface and legs blue-green with green tinge, pronotum with two, weakly defined black spots, humeri and posterior half of elytra bronze with red lustre, ventral surface blue-green; antennae black with strong green lustre.

Head large, as wide as anterior pronotal margin; clypeus trapezoidal, separated from frons by deep, transverse depression, anterior margin rounded; frons deeply, widely grooved, vertex almost flat, about twice as wide as width of eye; antennae slightly extending beyond midlength of lateral pronotal margins when laid alongside, antennomeres 5-10 widely trapezoidal; sculpture of head consisting of small, oval cells with large, flat central grains which are dense along eyes and very sparse in middle.

Pronotum convex, 2.2 times as wide as long, posterior margin deeply biarcuate; lateral margins slightly S-shaped, posterior angles sharp; “agriloid” carina well defined, reaching posterior third of lateral margins; lateroposterior depressions and basal tubercles weakly defined, prescutellar pit large, deep; sculpture consisting of basal microsculpture and dense, weakly defined polygonal cells without central grains. Scutellum cordiform, somewhat longer than wide, convex.

Elytra regularly convex, twice as long as wide, with obtusely rounded apices, not caudiform; humeral swellings small, basal, transverse depression deep, wide, reaching scutellum; elytral epipleura well defined, subparallel, reaching elytral apex; subhumeral carina strongly defined, nearly reaching elytral apex; sculpture consisting of short, transverse, zig-zag rugae.

Ventral surface roughly ocellate, prosternum slightly convex with shallow transverse groove just behind anterior margin which is in this way transformed into fine, transverse, perpendicular ledge (as in Fig. 34e); both anal ventrite and tergite simply rounded, not serrate. Legs moderately long, all tarsi shorter than tibiae; tarsal claws strong, simply hook-shaped.

Male unknown.

##### Etymology.

The subgenus *Paraguayetta* subgen. n. (feminine) is named after the country of the origin (Paraguay).

##### Differential diagnosis.

The subgenus *Paraguayetta* subgen. n. differs from other subgenera of *Bilyaxia* by the large, robust body, anteriorly rounded clypeus, S-shaped lateral, pronotal margins, weakly defined lateroposterior pronotal depressions, large and deep prescutellar pit, ledge-shaped anterior prosternal margin (as in Fig. 34e) and by the simple, not serrate anal ventrite.

##### Species included.

*Bilyaxia (Paraguayetta) mariae* (Cobos, 1956).

##### Note.

Except for the holotype (female, MNCN), only one further specimen was found in the collections: the female holotype of *Brasilaxia jacobi* Obenberger, 1958 which was synonymized with *Anthaxia (Cylindrophora) mariae* by Cobos (1972). This specimen (female, NMPC) is labelled: “Paraguay, Alto Paraná, Hohenau, H. Jacob[p], Nov. 1931[h]”.

#### Bilyaxia (Tomasia) subgen. n.

Figs 13–15

##### Type species.

*Anthaxia maculicollis* Kerremans, 1887 by present designation.

##### Diagnosis.

Small to medium sized (3.3–4.8 mm), slender, subcylindrical species.

Head relatively large, slightly wider or as wide as anterior pronotal margin; clypeus wide, trapezoidal, anterior margin straight or weakly emarginate; frons flat or convex, rarely weakly grooved; vertex flat or weakly convex, 1.8–2.2 times as wide as width of eye; eyes large, reniform, often slightly projecting beyond outline of head; sculpture of head consisting of small, fine, oval or polygonal cells.

Pronotum subcylindrical or lateral margins regularly rounded, 1.8–2.0 times as wide as long (exceptionally lateral margins S-shaped: *Bilyaxia (Tomasia) hayeki* (Cobos, 1972)); lateroposterior depressions wide, well defined; “agriloid” carina fine but well defined, usually reaching posterior third of lateral margins; prescutellar pit missing, basal tubercles weakly defined or missing; pronotal sculpture consisting of fine, poorly defined, polygonal cells without central grains and usually also by very fine basal microsculpture. Scutellum small to very small, cordiform, longer than wide.

Elytra regularly convex, smooth or with more or less defined, longitudinal costae, 2.2–2.4 times as long as wide; humeral swellings small but well defined, basal transverse depressions usually wide, deep, rarely shallow but always reaching scutellum; elytral epipleura narrowing posteriorly, reaching elytral apex; each elytron narrowly rounded, sometimes slightly caudiform; subhumeral carina well defined, usually reaching apical portion of elytra, sometimes shortened, scarcely reaching elytral midlength.

Anal ventrite usually simply rounded, finely serrate. Legs slender, long, tarsi not enlarged, distinctly shorter than tibiae; tarsal claws short, slightly curved.

##### Etymology.

The subgenus *Tomasia* subgen. n. (feminine) is named after my friend and colleague Tomás Moore Rodríguez (Santiago, Chile), the well-known specialist in the taxonomy of Chilean Buprestidae.

##### Species included.

*Bilyaxia (Tomasia) bruchiana* (Obenberger, 1926), *Bilyaxia (Tomasia) cinctipennis* (Kerremans, 1913), *Bilyaxia (Tomasia) cyaneoviridis* (Kerremans, 1900), *Bilyaxia (Tomasia) emmanueli* (Cobos, 1972), *Bilyaxia (Tomasia) hayeki* (Cobos, 1972), *Bilyaxia (Tomasia) lata* (Kerremans, 1903) and *Bilyaxia (Tomasia) macullicollis* (Kerremans, 1887).

##### Note.

*Bilyaxia (Tomasia) cinctipennis* (Fig. 15) somewhat contradicts the definition of the subgenus by its colouration (black and yellow pattern) and by the widely rounded elytral apices. In 2010 I found in Paraguay (Prov. Presidente Hayes) a specimen with entirely yellow elytra.

#### Brasilaxia

Théry, 1935

http://species-id.net/wiki/Brasilaxia

Fig. 16

##### Type species.

*Brasilaxia morretesi* Théry, 1935 by original designation.

The monotypic genus lacks taxonomic problems. The type species was synonymised with *Anthaxia costifera* Obenberger, 1913 by Cobos (1956). A further two species described in the genus *Brasilaxia* (*Brasilaxia jacobi* Obenberger, 1958 and *Brasilaxia olavei* Obenberger, 1958) were removed from *Brasilaxia* and synonymised by Cobos (1972) with *Anthaxia (Cylindrophora) mariae* (*Brasilaxia jacobi*) – see above, and with *Curis oyarcei* Germain & Kerremans, 1906 (*Brasilaxia olavei*–currently *Ctenoderus oyarcei* in Curidini).

##### Species included.

*Brasilaxia costifera* (Obenberger, 1913).

#### Paracuris

Obenberger, 1923

http://species-id.net/wiki/Paracuris

Figs 17, 18

##### Type species.

*Curis hemiptera* Burmeister, 1872 by original designation.

This monotypic genus lacks taxonomic problems and has very often been treated as a subgenus of *Anthaxia*, e.g. Bellamy (1985, 2003), Bílý (1997), Cobos (1956, 1986). Bílý (2004) upgraded it to genus which was was accepted by Bellamy (2008).

The type species, *Curis hemiptera*, was synonymized with *Anthaxia bimaculata* Gory, 1841 by Berg (1884) and Cobos (1956) described the subspecies *Anthaxia (Paracuris) bimaculata litigiosa* Cobos, 1956 from Argentina (Rio Colorado, Rio Negro).

##### Note.

The nominotypical subspecies possesses rather strong sexual dichromatism (Figs 17, 18). The female is rather similar to *Paracuris bimaculata litigiosa* and only the different form of the male genitalia (Figs 45–48 in Cobos, 1956) justifies treating the subspecies *litigiosa* as a valid taxon.

#### Anilaroides

Théry, 1934

http://species-id.net/wiki/Anilaroides

Fig. 26

##### Type species.

*Anilara brasiliensis* Kerremans, 1900 by original designation.

The genus was originally described by Théry (1934) as a subgenus of *Tetragonoschema* Thompson, 1857 which was accepted also by Bellamy (2008). The subgenus was subsequently upgraded to a genus by Bílý (2012). The genus *Anilaroides* contains only two very similar species distributed in Brasil and revised by Bílý (2012).

#### Tetragonoschema

Thomson, 1857

http://species-id.net/wiki/Tetragonoschema

Figs 27, 28

##### Type species.

*Tetragonoschema chrysomelinum* Thomson, 1857 (by monotypy; currently junior subjective synonym of *Tetragonoschema quadratum* (Buquet, 1841)).

The genus contains 17 species distributed from Mexico to Patagonia. The genus was quite recently revised by Bílý (2012) and divided into two subgenera.

#### Tetragonoschema (Tetragonoschema) s. str.

Fig. 27

##### Remarks.

The nominotypical subgenus contains 14 black, bronze or multicolorous, shortened (sometimes nearly as wide as long) species; elytra usually very short, flat, conspicuously uneven with one common, medial depression and with deep lateral and preapical depressions; elytral epipleura wide, well-developed, reaching or nearly reaching elytral suture; frons usually deeply impressed with projecting prominences above antennal insertions, rarely frons convex; pronotum weakly convex, transverse, usually with more or less distinct lateroposterior depressions; antennae and tarsi black; aedeagus spindle-shaped or elongate, parameres very often with lateral hooks or spines. The subgenus is distributed from Mexico to central Argentina.

#### Tetragonoschema (Patagoschema)

Bílý, 2012

Fig. 28

##### Type species.

*Tetragonoschema patagonicum* Obenberger, 1922 by original designation.

The subgenus contains 3 dark bronze, subcylindrical species; elytra completely or partly red-bronze, convex, without lateral and preapical depressions only with flat, medial, triangular depression at anterior elytral third; elytral epipleura narrow, not reaching elytral apex; frons convex with weak postclypeal depression; pronotum regularly, rather strongly convex, sometimes with two small, weakly developed, rounded depressions; antennae and tarsi reddish-brown; aedeagus short, spindle-shaped. The subgenus is distributed in southern Argentina (Patagonia).

##### Note.

*Tetragonoschema (Patagoschema) patagonicum* (Fig. 28) is the southernmost distributed species of the genus *Tetragonoschema* (Patagonia, Santa Cruz). The province of Santa Cruz is situated south of latitude 46 where the climatic conditions are rather extreme and *Tetragonoschema (Patagoschema) patagonicum* is most probably one of the southernmost distributed species of the Neotropical Buprestids.

## Supplementary Material

XML Treatment for Anthaxita

XML Treatment for Anthaxita peruviana

XML Treatment for Charlesina

XML Treatment for Charlesina mrazi mrazi

XML Treatment for Cobosina

XML Treatment for Cobosina willineri

XML Treatment for Marikia

XML Treatment for Marikia descarpentriesi

XML Treatment for Sanchezia

XML Treatment for Sanchezia bucki

XML Treatment for Agrilaxia

XML Treatment for Agrilaxia (Costiptera)

XML Treatment for Agrilaxia (Agrilaxia)

XML Treatment for Bilyaxia

XML Treatment for Bilyaxia (Bilyaxia)

XML Treatment for Bilyaxia (Paraguayetta)

XML Treatment for Bilyaxia (Tomasia)

XML Treatment for Brasilaxia

XML Treatment for Paracuris

XML Treatment for Anilaroides

XML Treatment for Tetragonoschema

XML Treatment for Tetragonoschema (Tetragonoschema)

XML Treatment for Tetragonoschema (Patagoschema)
